# Dynamics of a methanol-fed marine denitrifying biofilm: 1-Impact of environmental changes on the denitrification and the co-occurrence of *Methylophaga nitratireducenticrescens* and * Hyphomicrobium nitrativorans*

**DOI:** 10.7717/peerj.7497

**Published:** 2019-08-13

**Authors:** Geneviève Payette, Valérie Geoffroy, Christine Martineau, Richard Villemur

**Affiliations:** 1 INRS-Centre Armand-Frappier Santé et Biotechnologie, Laval, Québec, Canada; 2 Lallemand, Montréal, Québec, Canada; 3 Laurentian Forestry Centre, Québec, Québec, Canada

**Keywords:** Denitrification, Biofilm, Methylotrophy, *Methylophaga*, *Hyphomicrobium*, Co-occurrence

## Abstract

**Background:**

The biofilm of a methanol-fed denitrification system that treated a marine effluent is composed of multi-species microorganisms, among which * Hyphomicrobium nitrativorans* strain NL23 and *Methylophaga nitratireducenticrescens* strain JAM1 are the principal bacteria involved in the denitrifying activities. Here, we report the capacity of the denitrifying biofilm to sustain environmental changes, and the impact of these changes on the co-occurrence of * H. nitrativorans* and * M. nitratireducenticrescens*.

**Methods:**

In a first set of assays, the original biofilm (OB) was cultivated in an artificial seawater (ASW) medium under anoxic conditions to colonize new carriers. The new formed biofilm was then subjected to short exposures (1–5 days) of a range of NaCl, methanol, nitrate (NO_3_^−^) and nitrite (NO_2_^−^) concentrations, and to different pHs and temperatures. In a second set of assays, the OB was cultivated in ASW medium for five weeks with (i) a range of NaCl concentrations, (ii) four combinations of NO_3_^−^/methanol concentrations and temperatures, (iii) NO_2_^−^, and (iv) under oxic conditions. Finally, the OB was cultivated for five weeks in the commercial Instant Ocean (IO) seawater. The growth of the biofilm and the dynamics of NO_3_^−^ and NO_2_^−^ were determined. The levels of * M. nitratireducenticrescens* and * H. nitrativorans* were measured by qPCR.

**Results:**

In the first set of assays, the biofilm cultures had the capacity to sustain denitrifying activities in most of the tested conditions. Inhibition occurred when they were exposed to high pH (10) or to high methanol concentration (1.5%). In the second set of assays, the highest specific denitrification rates occurred with the biofilm cultures cultivated at 64.3 mM NO_3_^−^ and 0.45% methanol, and at 30 °C. Poor biofilm development occurred with the biofilm cultures cultivated at 5% and 8% NaCl. In all biofilm cultures cultivated in ASW at 2.75% NaCl, *H. nitrativorans* strain NL23 decreased by three orders of magnitude in concentrations compared to that found in OB. This decrease coincided with the increase of the same magnitude of a subpopulation of *M. nitratireducenticrescens* (strain GP59 as representative). In the biofilm cultures cultivated at low NaCl concentrations (0% to 1.0%), persistence of *H. nitrativorans* strain NL23 was observed, with the gradual increase in concentrations of *M. nitratireducenticrescens* strain GP59. High levels of *H. nitrativorans* strain NL23 were found in the IO biofilm cultures. The concentrations of* M. nitratireducenticrescens* strain JAM1 were lower in most of the biofilms cultures than in OB.

**Conclusions:**

These results demonstrate the plasticity of the marine methylotrophic denitrifying biofilm in adapting to different environmental changes. The NaCl concentration is a crucial factor in the dynamics of *H. nitrativorans* strain NL23, for which growth was impaired above 1% NaCl in the ASW-based biofilm cultures in favor of *M. nitratireducenticrescens* strain GP59.

## Introduction

Most naturally-occurring microbial biofilms, such as those encountered in bioremediation processes, are composed of multiple microbial species. Studying such complex biofilms is a challenge, as each species can influence the biofilm development. The biofilm microbial community inside a bioremediation process adapts to the prescribed operating conditions and shapes the efficiency of the bioprocess to degrade the pollutant(s) (Cabrol & Malhautier, 2011; Roder, Sorensen & Burmolle, 2016; Salta et al., 2013; Tan et al., 2017). The mechanisms of how a microbial population in a biofilm adapts to changes however are poorly understood.

Denitrification takes place in bacterial cells where N oxides serve as terminal electron acceptors instead of oxygen (O_2_) for energy production when oxygen depletion occurs, leading to the production of gaseous nitrogen (N_2_). Four sequential reactions are strictly required for the reduction of NO_3_^−^ to gaseous nitrogen, via nitrite (NO_2_^−^), nitric oxide and nitrous oxide, and each of these reactions is catalyzed by different enzymes, namely NO_3_^−^ reductases (Nar and Nap), NO_2_^−^ reductases (Nir), nitric oxide reductases (Nor) and nitrous oxide reductases (Nos) (Kraft, Strous & Tegetmeyer, 2011; Philippot & Hojberg, 1999; Richardson et al., 2001). These biological activities have been used with success to remove NO_3_^−^ in different wastewater treatment processes (Tchobanoglous, Burton & Stensel, 2003). The Montreal Biodome, a natural science museum, operated a continuous, fluidized-bed methanol-fed denitrification reactor to remove NO_3_^−^ that accumulated (up to 200 mg NO_3_^−^-N/L) in the 3 million-L seawater aquarium that contains fish, birds and invertebrates. The fluidized carriers in the denitrification reactor were colonized by naturally occurring multispecies bacteria to generate a marine methylotrophic denitrifying biofilm to be composed of 15–20 bacterial species (Labbé et al., 2003). Fluorescence *in situ* hybridization experiments on the biofilm showed that the methylotrophic bacteria *Methylophaga* spp. and *Hyphomicrobium* spp. accounted for 60 to 80% of the bacterial community (Labbé et al., 2007).

*Hyphomicrobium* spp. are methylotrophic bacteria that are ubiquitous in the environment (Gliesche, Fesefeld & Hirsch, 2005). They have also been found in significant levels in several methanol-fed denitrification systems treating municipal or industrial wastewaters or a seawater aquarium, and they occurred often with other denitrifying bacteria such as *Paracoccus* spp., *Methylophilales* or *Methyloversatilis* spp*.* Their presence correlates with optimal performance of bioprocess denitrifying activities (Baytshtok et al., 2008; CatalanSakairi, Wang & Matsumura, 1997; Ginige et al., 2004; Layton et al., 2000; Lemmer et al., 1997; Rissanen et al., 2017; Wang et al., 2014). *Methylophaga* spp. are methylotrophic bacteria isolated from saline environments (Boden, 2012; Bowman, 2005). They have been found in association with diatoms, phytoplankton blooms, marine algae, which are known to generate C1 carbons (Bertrand et al., 2015; Landa et al., 2018; Li et al., 2007). The importance of the co-occurrence of *Methylophaga* spp. and *Hyphomicrobium* spp. has been shown in two other methanol-fed denitrification systems treating saline effluents (Osaka et al., 2008; Rissanen et al., 2016). Therefore, understanding how collaboration between these two taxa could benefit in optimizing denitrification systems.

Two bacterial strains representatives of *Methylophaga* spp. and *Hyphomicrobium* spp. were isolated from the Biodome denitrifying biofilm. The first one, *Methylophaga nitratireducenticrescens* strain JAM1, is capable of growing in pure cultures under anoxic conditions by reducing NO_3_^−^ to NO_2_^−^, which accumulates in the medium (Auclair et al., 2010; Villeneuve et al., 2013). It was later shown to be able to reduce NO and N_2_O to N_2_ (Mauffrey et al., 2017). The second bacterium, *Hyphomicrobium nitrativorans* strain NL23, is capable of complete denitrification from NO_3_^−^ to N_2_ (Martineau et al., 2013b). These two strains are the main bacteria responsible of the dynamics of denitrification in the biofilm. The genomes of *M. nitratireducenticrescens* strain JAM1 and *H. nitrativorans* strain NL23 have been sequenced previously (Martineau et al., 2013a; Villeneuve et al., 2012). Strain JAM1 contains two Nar-type NO_3_^−^ reductase gene clusters (Nar1 and Nar2). Both Nar systems contribute to the reduction of NO_3_^−^ to NO_2_^−^ during growth of strain JAM1 (Mauffrey, Martineau & Villemur, 2015). Gene clusters encoding two Nor and one Nos systems are present and expressed in strain JAM1. Their presence correlates with the reduction of NO and N_2_O by strain JAM1 (Mauffrey et al., 2017). A dissimilatory NO-forming NO_2_^−^ reductase gene (*nirS* or *nirK*) is absent, which correlates with accumulation of NO_2_^−^ in the culture medium during NO_3_^−^ reduction. The genome of *H. nitrativorans* strain NL23 contains the operons that encode for the four different nitrogen oxide reductases, among which a Nap-type NO_3_^−^ reductase (Martineau, Mauffrey & Villemur, 2015).

We have initiated a study with the aim of assessing the performance of the Biodome denitrifying biofilm when exposed to environmental changes. The original biofilm (OB) taken from the Biodome denitrification system was cultivated in a homemade artificial seawater (ASW) medium, instead of the commercial Instant Ocean (IO) used by the Biodome, under batch mode and anoxic conditions at laboratory scale and exposed to a range of specific physico-chemical parameters. Thus, the objectives of this study were to determine the impact of these changes: (1) on the denitrification performance of the biofilm; (2) on the dynamics of the co-occurrence of the *H. nitrativorans* and *M. nitratireducenticrescens* in the biofilm; and (3) on the overall microbial community. The fourth objective of the study was to determine whether denitrifying bacteria other than *H. nitrativorans* strain NL23 and *M. nitratireducenticrescens* strain JAM1 are present in the biofilm.

In the first part of this study reported by Geoffroy et al. (2018), we showed that by cultivating the OB in the ASW medium, important decreases in the concentration of *H. nitrativorans* strain NL23 occurred without the loss of denitrifying activities. This decrease concurred with the occurrence of the new denitrifying bacteria *M. nitratireducenticrescens* strain GP59. The genome of strain GP59 has been sequenced and is highly similar to that of strain JAM1. Both strains have the same denitrification genes except that the genome of strain GP59 contains a *nirK* gene, which is missing in strain JAM1 (Geoffroy et al., 2018).

Here, we report the results for the first two objectives of our study. First, the OB was cultivated in ASW medium under anoxic conditions to derive the reference biofilm cultures. These cultures were then exposed for short periods (1 to 5 days) to different physico-chemical parameters. Such parameters included a range of NaCl, NO_3_^−^, NO_2_^−^ and methanol concentrations, and varying pH and temperature. These parameters were chosen as possible factors that could affect a denitrification reactor. The OB was then cultivated for a long period (five weeks) under specific conditions based on the short exposure assays to develop new biofilm cultures. The impact of these changes on the denitrifying activities and on the population dynamics of *M. nitratireducenticrescens* strain JAM1 and strain GP59, and *H. nitrativorans* strain NL23 were measured. Results from the third and fourth objectives are presented by Villemur et al. (2019), where we have looked at the overall microbial community of our biofilm cultures and identified new denitrifying bacteria. To our knowledge, this is the first systematic study on the co-evolution of *Methylophaga* and *Hyphomicrobium* strains in a marine biofilm system.

**Table 1:** Incubation conditions of the reference biofilm cultures (Ref 300N-23C) exposed for a short period to specific environmental conditions.

Name	NO_3_^−^/NO_2_^−^ mg-N/L (mM)	Methanol % (v/v)	C/N	NaCl % (w/v)	pH	Temp C
Tested conditions						
NO_3_^−^ and methanol (C/N=1.5). Exposure: 12–48 h						
*300*_*N*−*NO*3_*-0.15%*_*MeOH*_^a^	*300* (*21.4*)	*0.15*	*1.5*	*2.75*	*8.0*	*23*
600_*N*−NO3_-0.3%_MeOH_	600 (42.8)	0.3	1.5	2.75	8.0	23
900_*N*−NO3_-0.45%_MeOH_	900 (64.3)	0.45	1.5	2.75	8.0	23
1500_*N*−NO3_-0.75%_MeOH_	1500 (107)	0.75	1.5	2.75	8.0	23
3000_*N*−NO3_-1.5%_MeOH_	3000 (214)	1.5	1.5	2.75	8.0	23
NO_2_^−^. Exposure: 12–48 h						
400_*N*−NO2_	400 (28.6)	0.15	1.5	2.75	8.0	23
200_*N*−NO2_	200 (14.3)	0.15	1.5	2.75	8.0	23
200_N_-_NO3_/200_*N*−NO2_	200+200 (28.6)	0.15	1.5	2.75	8.0	23
Methanol (C/N variable). Exposure: 12–48 h						
0%_MeOH_	300 (21.4)	0	0	2.75	8.0	23
0.05%_MeOH_	300 (21.4)	0.05	0.5	2.75	8.0	23
*0.15%*_*MeOH*_^a^	*300* (*21.4*)	*0.15*	*1.5*	*2.75*	*8.0*	*23*
0.5%_MeOH_	300 (21.4)	0.5	5	2.75	8.0	23
NO_3_^−^. (C/N variable): Exposure: 12–48 h						
90_*N*−NO3_	90 (6.4)	0.15	5	2.75	8.0	23
*300*_*N*−NO3_^a^	300 (*21.4*)	*0.15*	*1.5*	*2.75*	*8.0*	*23*
900_*N*−NO3_	900 (64.3)	0.15	0.5	2.75	8.0	23
3000_*N*−NO3_	3000 (214)	0.15	0.15	2.75	8.0	23
pH**. Exposure 48–120 h						
pH4	300 (21.4)	0.15	1.5	2.75	4.0	23
pH6	300 (21.4)	0.15	1.5	2.75	6.0	23
pH8*	*300* (*21.4*)	*0.15*	*1.5*	*2.75*	*8.0*	*23*
pH10	300 (21.4)	0.15	1.5	2.75	10.0	23
Temperature**. Exposure 48–120 h						
5 °C	300 (21.4)	0.15	1.5	2.75	8.0	5
15 °C	300 (21.4)	0.15	1.5	2.75	8.0	15
23 °C*	*300 (21.4)*	*0.15*	*1.5*	*2.75*	*8.0*	*23*
30 °C	300 (21.4)	0.15	1.5	2.75	8.0	30
36 °C	300 (21.4)	0.15	1.5	2.75	8.0	36
NaCl**. Exposure 48–120 h						
0%_NaCl_	300 (21.4)	0.15	1.5	0	8.0	23
1%_NaCl_	300 (21.4)	0.15	1.5	1.0	8.0	23
*2.75%*_*NaCl*_^a^	*300 (21.4)*	*0.15*	*1.5*	*2.75*	*8.0*	*23*
5%_NaCl_	300 (21.4)	0.15	1.5	5.0	8.0	23
8%_NaCl_	300 (21.4)	0.15	1.5	8.0	8.0	23

**Notes.**

The ASW medium was adjusted for specific concentrations of methanol (MeOH), NO_3_^−^, NO_2_^−^ and NaCl, and pH. The biofilm carriers from the reference biofilm cultures were transferred in these solutions and incubated under the prescribed temperature. The 200_N-NO3-_200_N-NO2_ assays were done with a mix of 200 mg NO_3_^−^-N/L and 200 mg NO_2_^−^-N/L. In gray are parameters different from those of the reference biofilm cultures. C/N: Carbon Nitrogen ratio. A 1.5 C/N ratio corresponds, for instance, to 300 mg NaNO_3_-N/L (21.4 mM) and 450 mg CH3OH-C/L (37.5 mM or 0.15% v/v). All assays were carried out in triplicates, except the NO2-assays (one replicate). See Fig. S2 for more details.

a Reference biofilm cultures.

b The reference biofilm cultures were incubated in the prescribed conditions for two days, and then transferred in fresh medium for 12 to 72 h.

## Material and Methods

### Seawater media

The artificial seawater (ASW) medium was composed of (for 1 liter solution): 27.5 g NaCl, 10.68 g MgCl_2_*6H_2_O, 2 g MgSO_4_*7H_2_O, 1 g KCl, 0.5 g CaCl_2_, 456 µL of FeSO_4_*7H_2_O 4 g/L, five mL of KH_2_PO_4_51.2 g/L, five mL of Na_2_HPO_4_ 34 g/L. The Instant Ocean (IO) seawater medium was bought from Aquarium systems (Mentor, OH, USA) and dissolved at 30 g/L. Estimation of its composition and comparison with the ASW medium is provided in Table S1. Both media were supplemented with one mL of trace elements (FeSO_4_*7H_2_O 0.9 g/L, CuSO_4_*5H_2_O 0.03 g/L and MnSO_4_*H_2_O 0.2 g/L) and with NaNO_3_ (Fisher Scientific Canada, Ottawa, ON, Canada) at various concentrations, depending on the experiment (Tables 1 and 2). The pH was adjusted (NaOH) at 8.0 before autoclaving. Filter-sterilized methanol (Fisher Scientific) was then added as a carbon source to support bacterial growth, also at various concentrations depending on the experiment (Tables 1 and 2). The sterile media were distributed (60 ml) in sterile serologic vials, which included twenty carriers (Bioflow 9 mm; Rauschert, Steinwiessen, Allemagne). Prior to use, these carriers were washed with HCl 10% (v/v) for 3 h, rinsed with water and autoclaved. The vials were purged of oxygen for 10 min with nitrogen gas (Praxair, Mississauga, ON, Canada) and sealed with sterile septum caps.

**Table 2:** Culture conditions used for the cultivation of the original biofilm.

Name	Medium	NO_3_^−^/NO_2_^−^ mg-N/L (mM)	Methanol % (v/v)	NaCl % (w/v)	Temp °C
Ref300N-23C^a^	ASW	300 (21.4)	0.15	2.75	23
Oxic^b^	ASW	300 (21.4)	0.15	2.75	23
300N-30C	ASW	300 (21.4)	0.15	2.75	30
900N-23C	ASW	900 (64.3)	0.45	2.75	23
900N-30C	ASW	900 (64.3)	0.45	2.75	30
0%NaCl	ASW	300 (21.4)	0.15	0	23
0.5%NaCl	ASW	300 (21.4)	0.15	0.5	23
1%NaCl	ASW	300 (21.4)	0.15	1.0	23
5%NaCl	ASW	300 (21.4)	0.15	5.0	23
8%NaCl	ASW	300 (21.4)	0.15	8.0	23
2.75–5%NaCl^c^	ASW	300 (21.4)	0.15	2.75/5	23
IO	IO	300 (21.4)	0.15	3.0^d^	23
200-200N	ASW	200 NO_3_^−^/200 NO_2_^−^ (28.6)	0.15	2.75	23

**Notes.**

The original biofilm was cultivated in triplicates in these conditions at pH 8.0. The carriers were transferred five times in fresh medium (eight times for the 200-200N biofilm cultures). In gray are changed parameters from the Ref300N-23C biofilm cultures. IO: Instant Ocean medium.

a Reference biofilm cultures.

b Cultures were performed under oxic conditions.

c The Ref300N-23C biofilm cultures were further transferred three times in ASW composed of 5% NaCl.

d Based on the amount of Na^+^ and Cl^-^ in Table S1. The exact amount of NaCl added in the IO medium is not known. For comparison, the amount of Na^+^ and Cl^-^ in the ASW medium is 3.2%.

### The reference biofilm cultures (Ref300N-23C)

The carriers (Bioflow 9 mm) containing the denitrifying biofilm (here named the Original Biofilm; OB) were taken from the denitrification reactor of the Montreal Biodome and frozen at −20 °C in seawater with 20% glycerol (Laurin et al., 2006) until use. Biomass from several carriers were thawed, scraped, weighted and dispersed in the ASW at 0.08 g (wet weight)/mL. The biomass (five mL/vial, 0.4 g of biofilm) was then distributed with a syringe and an 18_G_1 $ \frac{1}{2} $ needle into vials containing twenty free carriers and 60 mL ASW and supplemented with 300 mg NO_3_^−^-N/L (21.4 mM NO_3_^−^) and 0.15% (v/v) methanol (C/N = 1.5). The vials were incubated at 23 °C and shaken at 100 rpm (orbital shaker).

On average once a week, the twenty carriers were taken out of the vial, gently washed to remove the excess medium and the free bacteria, transferred into a new vial containing fresh anaerobic medium, and then incubated again under the same conditions (Fig. S1). Samples were taken each one or two days to measure the concentrations of NO_3_^−^ and NO_2_^−^ in the growth medium. The residual suspended biomass was taken for DNA extraction. Methanol and NaNO_3_ were added when needed if NO_3_^−^ was completely depleted during the week. During the fifth carrier-transfer cultures, multiple samples (500 µL) were collected at regular intervals for 1–3 days to determine the NO_3_^−^ and NO_2_^−^ concentrations. After this monitoring, total biomass in a whole vial was determined by protein quantification.

### Short exposure of the reference biofilm cultures to physico-chemical changes

The carriers containing the reference biofilm cultures were used as starting material to test the impact on the denitrification performance of the biofilm by varying specific physico-chemical parameters (Table 1). Figure S2 describes in detail the protocol. To perform these assays, fifteen carriers from the reference biofilm cultures were distributed into vials in the prescribed conditions described in Table 1. For the assays that were performed with different pHs and temperatures and different NaCl concentrations, the vials were incubated for two days for the biofilm to adapt to the new culture conditions, and then the carriers were transferred into their respective fresh medium (Fig. S2). NO_3_^−^ and NO_2_^−^ concentrations in the vials were then monitored by collecting 500 µL medium samples at regular intervals for 1 to 3 days. Biomass was taken at the end of this monitoring to extract DNA and for protein quantification. All assays were performed in triplicates, except those with NO_2_^−^.

### Cultivation of the original biofilm to different culture conditions

The OB was taken and processed as described in *The reference biofilm cultures* subsection. The formulation of the ASW medium was adjusted with the prescribed NaCl, NO_3_^−^, NO_2_^−^ and methanol concentrations, and pHs, and the vials were incubated at prescribed temperatures (Table 2; Fig. S3). Cultivation of the OB under oxic conditions were performed as for the reference biofilm cultures but in 250-mL Erlenmeyer flasks with agitation (Table 2). Culturing the OB in the IO medium was performed as for the reference biofilm cultures (Table 2). All biofilm cultures were shaken at 100 rpm (orbital shaker), or 200 rpm for the oxic cultures. Five carrier-transfer cultures were performed, after which the NO_3_^−^, NO_2_^−^ and protein concentrations were determined as described in *The reference biofilm cultures* subsection.

### Determination of the denitrification rates

Measurements of NO_3_^−^ and NO_2_^−^ concentrations in the biofilm cultures were performed in all assays using ion chromatography as described by Mauffrey et al. (2017). The total biomass in a vial was measured by collecting the suspended biomass and the biomass attached to the carriers (Fig. S1 to Fig. S3). The amount of protein in the biomass was determined by the Quick Start™ Bradford Protein Assay (BioRad, Mississauga, ON, Canada). The denitrification rates were calculated by the linear portion of the NO_3_^−^ plus NO_2_^−^ concentrations (NO_x_) over time for each replicate (mM h^−1^). The specific denitrification rates were reported as the denitrification rates divided by the quantity of biomass (mg protein) in a vial (mM h^−1^ mg-protein^−1^). Ammonium concentration was measured using the colorimetric method described by Mulvaney (1996).

### DNA-based analyses

The DNA was extracted from the suspended biomass and the biofilm that was scraped from the carriers as previously described (Geoffroy et al., 2018). The PCR amplifications of the V3 region of the 16S ribosomal RNA (rRNA) genes for denaturating gradient gel electrophoresis (DGGE) experiments were performed as described (Lafortune et al., 2009) with the 341f and 534r primers (Table S2). Total DNA extracted from the planktonic pure cultures of strains JAM1 and NL23 was used to perform PCR amplifications of the same region of the 16S rRNA genes. The resulting strain-derived amplicons were co-migrated with the biofilm cultures-derived amplicons on DGGE gels to identify the DNA fragments associated to strain JAM1 and strain NL23 in biofilm samples.

Quantitative PCR assays (SYBR green) to measure the concentrations of *M. nitratireducenticrescens* strains JAM1/GP59 (*narG1*), *M. nitratireducenticrescens* strain GP59 (*nirK*), *M. nitratireducenticrescens* strain JAM1 (*tagH*) and *H. nitrativorans* strain NL23 (*napA*) (Table S2) in the biofilm cultures were performed as previously described (Geoffroy et al., 2018).

### Statistics

Statistical significance in the denitrification rates between different types of cultures was performed with one-way ANOVA with Tukey’s Multiple Comparison Test.

## Results

### Establishment of the reference biofilm cultures (Ref300N-23C)

The original biofilm (OB) of the Biodome denitrification reactor was used to inoculate vials containing new carriers to allow the development of a fresh denitrifying biofilm on the carrier surface. The vials were incubated under batch-mode anoxic conditions in a homemade ASW medium (2.75% NaCl) with 300 mg NO_3_^−^-N/L (21.4 mM NO_3_^−^) and 0.15% (v/v) methanol (C/N = 1.5) at 23 °C. The choice of ASW medium was based on our ability to change the composition of the medium, as opposed to the commercial seawater medium (Instant Ocean) used by the Biodome. Each week for five weeks, only the carriers were transferred in fresh medium (Fig. S1). The biofilm cultures cultivated under these conditions are referred as the *reference biofilm cultures* and named from here as the Ref300N-23C biofilm cultures (for 300 mg NO_3_^−^-N/L, 23 °C). As its name implies, the conditions of these cultures were used as a reference to measure changes in the denitrifying activities resulting from changes in physico-chemical parameters in the culture medium.

**Figure 1:**
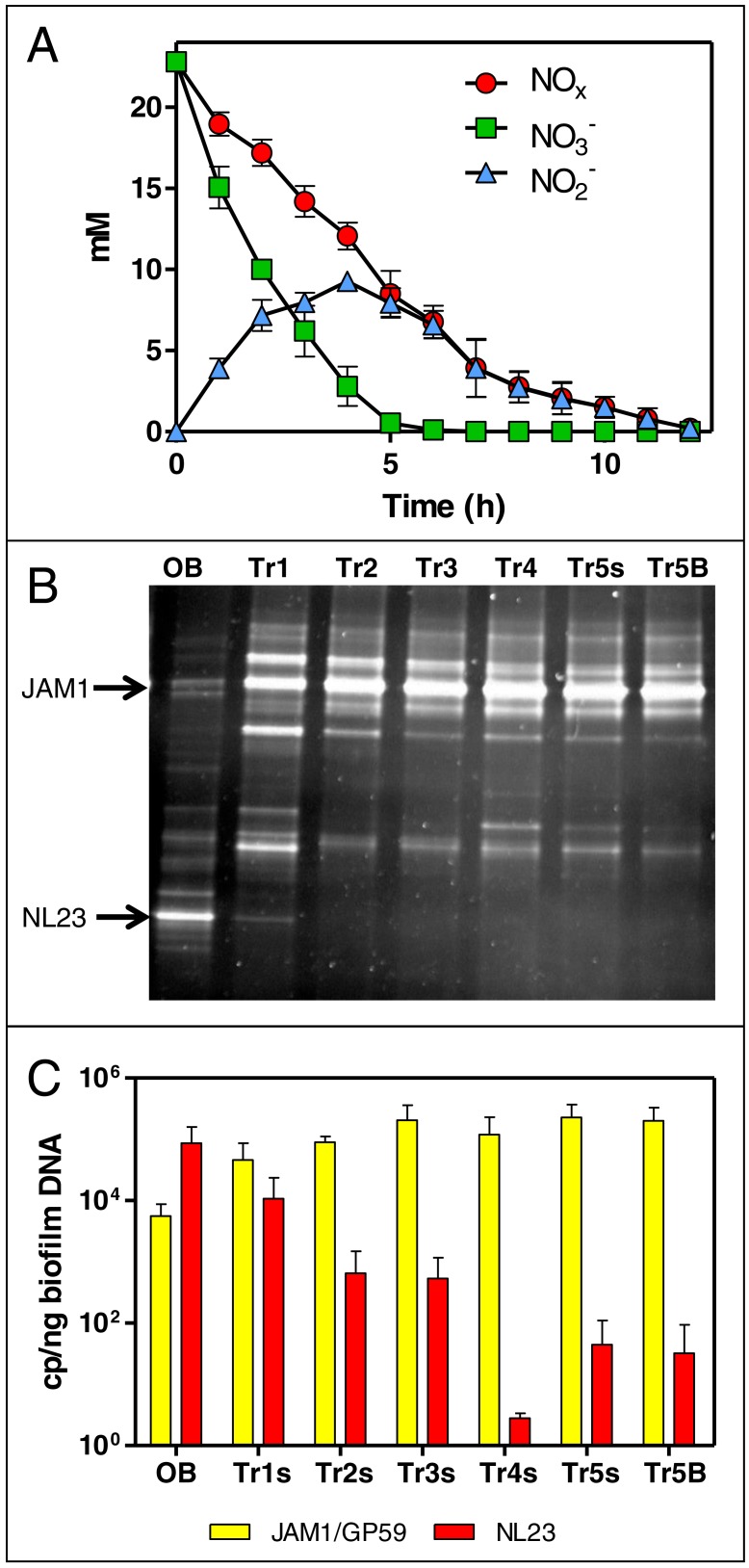
Dynamics of NO${}_{3}^{-}$ and NO${}_{2}^{-}$ concentrations and of the bacterial community in the Ref300N-23C biofilm cultures. (A) NO${}_{3}^{-}$ and NO${}_{2}^{-}$ concentrations were measured during the fifth carrier-transfer cultures. Results from triplicate biofilm cultures. (B) PCR-DGGE migration profiles representing the bacterial diversity during the first five carrier-transfer cultures. DNA extraction was performed on samples from the suspended biomass in the first four carrier-transfer cultures because the biofilm was not enough abundant on the carriers in these cultures. In the fifth carrier-transfer cultures, samples were taken from the suspended biomass (Tr5s) and the biofilm (Tr5B). OB: Original biofilm. Approximately 300 ng of the PCR product was loaded per sample. (C) Quantification of *M. nitratireducenticrescens* strains JAM1/GP59 (*narG1*) and * H. nitrativorans* strain NL23 (*napA*) by qPCR in the five carrier-transfer cultures (Tr1 to Tr5). Results from 3 to 9 replicate cultures, of one to three different inoculums.

The NO_3_^−^ and NO_2_^−^ concentrations were analyzed sporadically for the first four carrier-transfer cultures, and showed increasing rates of denitrifying activities with subsequent transfers. During the fifth carrier-transfer cultures, the concentrations of NO_3_^−^ and NO_2_^−^ were analyzed more thoroughly. NO_3_^−^ was consumed in 4 to 6 h, with accumulation of NO_2_^−^ that peaked at 10 mM after 4 h. NO_2_^−^ was completely consumed after 12 h (Fig. 1A). The denitrification rates were calculated at 1.69 (±0.14) mM-NO_x_ h^−1^. Taking into account the biomass, the specific denitrification rates corresponded to 0.0935 (±0.0036) mM-NO_x_ h^−1^ mg-protein^−1^. The production of ammonia was negative in these conditions, which rules out dissimilatory NO_3_^−^ reduction to ammonium. Further carrier transfers did not improve the specific denitrification rates.

The evolution of the bacterial community of the OB under these conditions was assessed by PCR-DGGE profiles for each carrier-transfer cultures (Fig. 1B). These profiles were similar after the third carrier-transfer cultures, suggesting stabilization of the bacterial community in these cultures. The most substantial changes in these profiles compared to the OB profile is the intensity of the DGGE band corresponding to *M. nitratireducenticrescens* strain JAM1 that was much stronger in all carrier-transfer cultures, whereas the DGGE band corresponding to *H. nitrativorans* strain NL23 was no longer visible after the second carrier-transfer cultures (Fig. 1B). qPCR analysis confirmed these results (Fig. 1C). Strain JAM1 increased in concentration in the Ref300N-23C biofilm cultures from 5.6 × 10^3^ *narG1* copies/ng DNA in the OB to 2.0 × 10^5^ *narG1* copies/ng DNA in the fifth carrier-transfer cultures. The concentrations of strain NL23 however dropped by three orders of magnitude from 8.7 × 10^4^ in the OB to 3.2 ×10^1^ *napA* copies/ng DNA in the fifth carrier-transfer cultures.

These results showed important changes in the bacterial community of OB occurred when cultivated in the ASW medium under the batch-operating mode, with the important decrease in concentration of the main denitrifying bacterium in the biofilm, *H. nitrativorans* strain NL23, without the loss of denitrifying activities. We discovered later that a new *M. nitratireducenticrescens* strain, strain GP59 with full denitrification capacity, was enriched in these cultures (Geoffroy et al., 2018). qPCR assays targeting *narG1* cannot discriminate strain JAM1 from strain GP59, as this gene is identical in nucleic sequence in both strains (Geoffroy et al., 2018). Therefore, the concentrations of strain JAM1 in the biomass derived from *narG1*-targeted qPCR assays and illustrated in Fig. 1C reflects in fact the concentrations of both strains.

### Impact of a short exposure to environmental changes on the Ref300N-23C biofilm cultures

The colonized carriers from the Ref300N-23C biofilm cultures were exposed for a short period (few hours to few days) to a range of methanol, NO_3_^−^, NO_2_^−^ and NaCl concentrations, and to different pHs and temperatures (Table 1, Fig. S2, Table S3). These conditions were chosen to assess the capacity range of the denitrifying activities of the biofilm (methanol and NO_3_^−^ concentrations), but also its resilience to adverse conditions that could occur during the operation of a bioprocess (e.g., abrupt change of pH, temperature or salt concentration).

**Figure 2:**
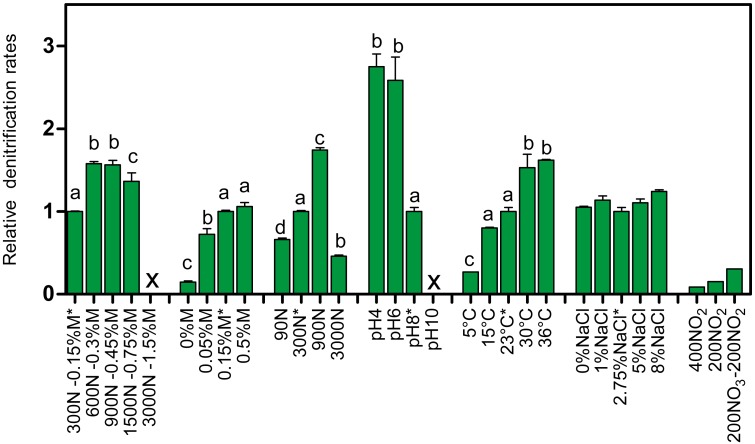
Denitrifying activities of the Ref300N-23C biofilm cultures impacted by different physico-chemical parameters. The Ref300N-23C biofilm cultures were exposed for few hours or few days in modified ASW medium as described in Table 1. Denitrification rates are expressed relative to the denitrification rates of the original conditions of the Ref300N-23C biofilm cultures (set to one) identified by *. Significance between the denitrification rates were determined by one-way ANOVA. Rates with the same letter are not significantly different. X: no activities recorded. Average of triplicates cultures except for the 400N-NO_2_, 200N-NO_2_ and 200N-NO_3_/200N-NO_2_ conditions with one replicate. N: Nitrogen NO${}_{3}^{-}$. M: methanol.

Increasing the concentrations of NO_3_^−^ and methanol (fixed C/N at 1.5) showed increases in the denitrification rates compared to the original conditions of the Ref300N-23C biofilm cultures (300 mg-NO_3_^−^-N/L with 0.15% methanol), with the highest increases observed at 600 mg-NO_3_^−^-N/L with 0.3% methanol (58% increase), and at 900 mg-NO_3_^−^-N/L with 0.45% methanol (56% increase) (Fig. 2). Inhibition of the denitrifying activities occurred at 3000 mg-NO_3_^−^-N/L. This inhibition could have been caused by the methanol concentration (1.5%) in the medium. Variations in methanol concentrations (0% to 0.5% with fixed concentration of NO_3_^−^ at 300 mg-NO_3_^−^-N/L) showed no improvement of the denitrification activities compared to the original conditions (Fig. 2). As methanol is the only source of carbon in the medium, its absence (0%) resulted in very weak denitrifying activities as expected. Variations in NO_3_^−^ concentrations (fixed concentration of methanol at 0.15%) showed the highest denitrification rates (74% increase) at 900 mg-NO_3_^−^-N/L (Fig. 2). Denitrifying activities were observed this time at 3000 mg-NO_3_^−^-N/L. However, NO_3_^−^ consumption stopped after 5 days and 50% consumption. Addition of methanol (0.15%) allowed activities to resume for one day with a further 20% NO_3_^−^ consumption.

Exposure of the Ref300N-23C biofilm cultures to pH 4 and 6 showed 2.7 and 2.6-fold increases of the denitrification rates, respectively, compared to the original conditions (pH 8) (Fig. 2). However, the pH at the end of the assays (10 to 12 h) increased at around 8 in the medium. At pH 10, the denitrifying activities were completely inhibited. For the temperature assays, the highest denitrification rates were recorded at 30 and 36 °C (Fig. 2), with 53% and 62% increases, respectively, compared to the original conditions (23 °C). At 5 °C, the denitrifying activities were still occurring but at 4-time lower rates than at 23 °C. At 15 °C, the denitrification rates were not significantly different from the original conditions. Exposure of the Ref300N-23C biofilm cultures for 3 days in ASW with NaCl concentrations ranging from 0% to 8% showed little effect on the denitrification rates (Fig. 2). Finally, presence of NO_2_^−^ has a strong effect on the denitrification rates with 11-fold and 6.6-fold decreases with 400 mg-NO_2_^−^-N/L and 200 mg-NO_2_^−^-N/L, respectively in the medium, compared to the original conditions (Fig. 2). Mixture of 200 mg-NO_2_^−^-N/L and 200 mg-NO_3_^−^-N/L in the medium had a less pronounce effect with a 3-fold decrease of the denitrification rates. Although these last three assays with NO_2_^−^ were performed with only one replicate, it revealed the tendency of NO_2_^−^ to affect negatively the denitrifying capacity of the biofilm.

No significant differences in the quantity of biomass (protein amount per vial) was observed at the end of all these assays between vials. In all the tested conditions, the bacterial diversity profiles (PCR-DGGE profiles) showed no changes (Fig. 1B, lane Tr5B). The levels of *M. nitratireducenticrescens* strains JAM1/GP59 was around 10^5^ *narG1* copies/ng DNA and of *H. nitrativorans* strain NL23 between 10^1^ and 10^−1^ *napA* copies/ng DNA.

### Cultivation of the original biofilm under optimal conditions

As described above, the Ref300N-23C biofilm cultures exposed for a short period to 900 mg-NO_3_^−^-N/L (0.45% methanol; C/N 1.5) or to 300 mg-NO_3_^−^-N/L (0.15% methanol; C/N 1.5) at 30 and 36 °C showed between 53% to 74% increases in the denitrification rates compared to the original conditions (Fig. 2). Based on these results, we cultivated the OB under these optimal conditions in the attempt to derive a biofilm with higher denitrifying capacity. As before, the dispersed biomass of the OB was used as inoculums to colonize new carriers. Five carrier-transfer cultures were carried out in ASW medium supplemented with 900 mg-NO_3_^−^-N/L and 0.45% methanol (C/N = 1.5), and incubated at either 23 °C or 30 °C (here named 900N-23C and 900N-30C, respectively), or at 300 mg-NO_3_^−^-N/L and 0.15% methanol (C/N = 1.5) at 30 °C (here named 300N-30C). In parallel, the Ref300N-23C biofilm cultures were derived by the same protocol (Table 2, Fig. S3). The specific denitrification rates of these biofilm cultures were 20% to 85% higher than those found in the Ref300N-23C biofilm cultures (Table 3). Assuming methanol was not a limiting factor in these cultures (not all methanol was consumed after NO_3_^−^ reduction), two-way ANOVA showed that temperature was the main factor affecting the specific denitrification rates (*p* < 0.001), not the concentrations of NO_3_^−^ (*p* > 0.05).

**Table 3:** Denitrifying activities in the biofilm cultures.

Biofilm cultures	Denitrification rates mM-NO_x_h^−1^ flask^−1^	Relative activities	Protein concentration mg/vial	Specific denitrification rates mM-NO_x_h^−1^ mg-protein^−1^	Relative specific activities
0%NaCl	1.28 (0.01)	0.87 (0.01)	14.1 (1.2)	0.0911 (0.0080)	1.72 (0.15)^b^
0.5%NaCl	1.57 (0.02)	1.06 (0.01)	22.2 (2.6)	0.0712 (0.0084)	1.34 (0.16)^ab^
1%NaCl	0.66 (0.01)	0.45 (0.01)	18.5 (0.2)	0.0357 (0.0011)	0.67 (0.02)^cd^
Ref300N-23C*	1.47 (0.05)	1.00 (0.03)	28.1 (0.4)	0.0530 (0.0064)	1.00 (0.12)^ad^
300N-30C	1.94 (0.04)	1.32 (0.03)	20.5 (0.9)	0.0946 (0.0027)	1.79 (0.05)^b^
900N-23C	1.95 (0.04)	1.32 (0.02)	30.7 (2.9)	0.0637 (0.0060)	1.20 (0.11)^abd^
900N-30C	2.37 (0.09)	1.61 (0.06)	24.5 (2.8)	0.0979 (0.0130)	1.85 (0.24)^b^
Ref300N-23C*	1.42 (0.01)	1.00 (0.01)	31.0 (1.8)	0.0458 (0.0027)	1.00 (0.06)^ad^
Oxic	0.18 (0.08)	0.12 (0.06)	19.8 (1.1)	0.0090 (0.0046)	0.20 (0.10)^c^
5%NaCl	0.06 (0.01)	0.04 (0.01)	0.38 (0.12)	0.1660 (0.0322)	3.63 (0.70)^e^
2.75–5%NaCl	2.46 (0.08)	1.74 (0.06)	62.7 (3.5)	0.0394 (0.0032)	0.86 (0.07)^ad^
8%NaCl	0.07 (0.01)	0.05 (0.01)	0.84 (0.04)	0.0845 (0.0071)	1.84 (0.15)^b^
Ref300N-23C*	1.89 (0.10)	1.00 (0.05)	27.6 (1.5)	0.0684 (0.0005)	1.00 (0.01)^ad^
IO	0.76 (0.03)	0.40 (0.01)	11.5 (0.8)	0.0661 (0.0063)	0.97 (0.10)^ad^
200/200N	0.39 (0.13)	0.41 (0.01)	nd	nd	nd

**Notes.**

Except for the 200/200N biofilm cultures, three sets of cultures assays were performed with the Ref300N-23C biofilm cultures in each set for comparison. Relative activities are to the denitrification rates of the Ref300N-23C biofilm cultures set to one. Protein concentrations reflect the biomass content in vials at the end of the assays. Specific denitrification rates are the denitrification rates divided by protein amount in vials. Relative specific activities are to the specific denitrification rates of the Ref300N-23C biofilm cultures set to one. Significance between the relative specific denitrification rates was determined by one-way ANOVA with Tukey’s Multiple Comparison Test. The relative specific denitrification rates with the same letter are not significantly different. See Table 2 and Material & Methods for nomenclature and culture conditions. Results are average of triplicate biofilm cultures. Values between parentheses: standard deviation. nd: not done.

**Figure 3:**
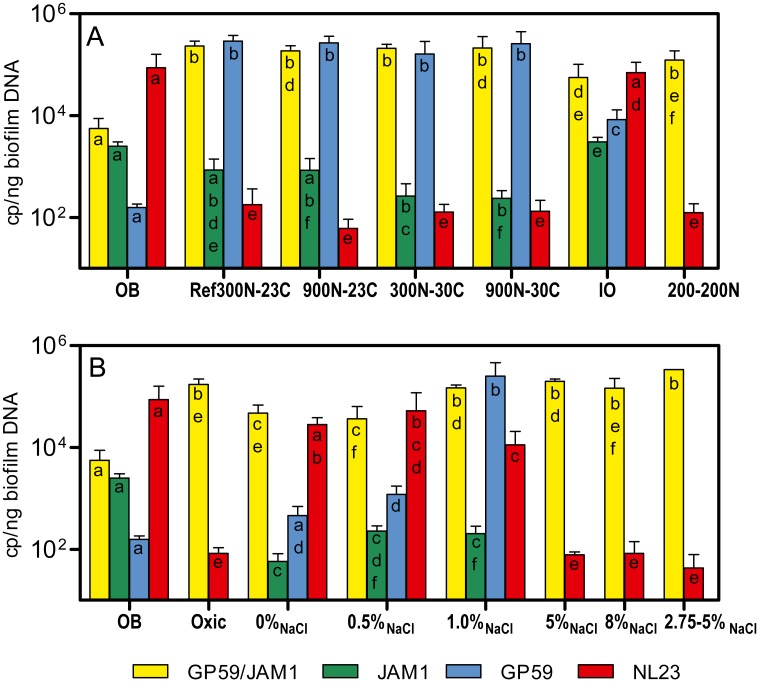
Denitrifying activities and dynamics of *H. nitrativorans* strain NL23 and * M. nitratireducenticrescens* in the biofilm cultures. (A, B) Quantification of *M. nitratireducenticrescens* strains JAM1/GP59 (*narG1*), * M. nitratireducenticrescens* strain JAM1 (*tagH*), * M. nitratireducenticrescens* strain GP59 (*nirK*) and * Hyphomicrobium nitrativorans* strain NL23 (*napA*) by qPCR in the corresponding biofilm cultures. Results from triplicate cultures. OB: Original biofilm. Significance of the qPCR levels (log_10_ transformed) of the respective genes between the biofilm cultures were determined by one-way ANOVA with Tukey’s Multiple Comparison Test. Rates with the same letter (within the bar) are not significantly different.

The bacterial diversity profiles (derived by PCR-DGGE) of these four biofilm cultures were all similar (same profile illustrated in Fig. 1B, lane Tr5B). qPCR analyses showed the same trend in the dynamics of the populations of *M. nitratireducenticrescens* strains JAM1/GP59 and *H. nitrativorans* strain NL23. The concentrations of strains JAM1/GP59 increased by two orders of magnitude (1.9 to 2.3 × 10^5^ *narG1* copies/ng DNA), whereas the concentrations of strain NL23 decreased by 2–3 orders of magnitude (6.1 to 18 × 10^1^ *napA* copies/ng DNA) in the four biofilm cultures compared to the OB (Fig. 3A). After the isolation of strain GP59 and the sequencing of its genome, qPCR primers specific to strain JAM1 and to strain GP59 were developed (Geoffroy et al., 2018). The concentrations of these two strains were then measured in the OB and in the four biofilm cultures. Between 1.6 and 2.9 ×10^5^ *nirK* copies/ng DNA of strain GP59 were measured in these biofilm cultures, which is three orders of magnitude higher than what was measured in the OB (1.6 ×10^2^ *nirK* copies/ng DNA). The concentrations of strain JAM1 in the four biofilm cultures ranged from 2.4 to 8.5  × 10^2^ *tagH* copies/ng DNA, which is 3 to 10 times lower than its concentration in the OB (2.5 ×10^3^ *tagH* copies/ng DNA) (Fig. 3A).

### Cultivation of the original biofilm with different NaCl concentrations

In the first set of assays, we showed that the Ref300N-23C biofilm cultures could sustain high variations of NaCl concentrations for a short period without affecting the denitrification capacity. Based on these results, we tested the capacity of the OB to acclimate to a range of NaCl concentrations. The dispersed biomass of the OB was cultivated during five carrier-transfers under anoxic conditions in modified ASW medium with NaCl concentrations ranging from 0% to 8% (Table 2, Fig. S3). The ASW used for the Ref300N-23C biofilm cultures contains 2.75% NaCl.

In biofilm cultures cultivated with low NaCl concentrations (0, 0.5 and 1.0%), the amounts of biomass developed were 1.3 to 2 times lower than in the Ref300N-23C biofilm cultures (Table 3). This lower development did not impair the specific denitrification rates of the 0% NaCl and 0.5% NaCl biofilm cultures, which increased by 72 and 34%, respectively, compared to the Ref300N-23C biofilm cultures. However, in the 1% NaCl biofilm cultures, these rates decreased by one third compared to the Ref300N-23C biofilm cultures (Table 3). The 5% NaCl and 8% NaCl biofilm cultures were strongly affected by poor biofilm development on the carriers, which was 50–100 less abundant in biomass than in the Ref300N-23C biofilm cultures (Table 3). In these biofilm cultures, NO_3_^−^ took more than 24 h to be consumed and NO_2_^−^ consumption was minimal. This result contradicts what was obtained in the first set of assays where the Ref300N-23C biofilm cultures subjected for 3 days to 5% and 8% NaCl did not affect the denitrification performance. To investigate this further, another Ref300N-23C biofilm cultures were derived, but after the fifth transfer cultures, the carriers were transferred in 5% NaCl ASW medium (here named 2.75–5% NaCl) and cultivated for another three carrier transfers (20 days). These biofilm cultures were able to sustain this high salt concentration. Compared to the Ref300N-23C biofilm cultures, the 2.75–5% NaCl biofilm cultures had a 74% increase in the denitrification rates. Because the biomass was twice the amount in these cultures, the specific denitrification rates were similar to those found in the Ref300N-23C biofilm cultures (Table 3).

The PCR-DGGE migrating profiles showed the persistence of strain NL23 in the 0% NaCl and 0.5% NaCl biofilm cultures. In the 1% NaCl biofilm cultures, however, strain NL23 was barely visible in DGGE profiles (Fig. S4). Persistence of strain NL23 was confirmed by qPCR where its concentrations in the 0% NaCl, 0.5% NaCl and 1.0% NaCl biofilm cultures (1.1 to 5.3 × 10^4^ *napA* cp/ng DNA) were at the same order of magnitude than in the OB (8.7 ×10^4^ *napA* cp/ng DNA) (Fig. 3B). As observed before, the concentrations of strain NL23 decreased by two to three orders of magnitude in the Ref300N-23C, 5% NaCl, 8% NaCl and 2.75–5% NaCl biofilm cultures (4.3 to 18 × 10^1^ *napA* cp/ng DNA) compared to the OB (Fig. 3B). The levels of *M. nitratireducenticrescens* strains JAM1/GP59 increased by one order of magnitude in the 0% NaCl and 0.5% NaCl biofilm cultures (*ca.* 4 × 10^4^ *narG1* cp/ng DNA) compared to the OB (5.6 × 10^3^ *narG1* cp/ng DNA), at similar levels than strain NL23 (Fig. 3B). However, in the 1% NaCl biofilm cultures, the concentrations of *M. nitratireducenticrescens* strains JAM1/GP59 increased by another order of magnitude (1.5 × 10^5^ *narG1* cp/ng DNA), which is similar to that found in the Ref300N-23C biofilm cultures (2.3 × 10^5^ *narG1* cp/ng DNA) (Figs. 3A and 3B). As observed before, the concentrations of strain JAM1 decreased by approximately one order of magnitude in these cultures (6 to 23 × 10^1^ *tagH* cp/ng DNA) when compared to the OB (2.5 × 10^3^*tagH* cp/ng DNA) (Fig. 3B). The concentrations of strain GP59 in the 0% NaCl biofilm cultures (4.6 × 10^2^ *nirK* cp/ng DNA) were at similar level as in the OB (1.6 × 10^2^ *nirK* cp/ng DNA). It increased by one order of magnitude in the 0.5% NaCl biofilm cultures (1.2 × 10^3^ *nirK* cp/ng DNA) and by two orders of magnitude in the 1.0% NaCl biofilm cultures (2.5 ×10^5^ *nirK* cp/ng DNA), similar to the levels reached in the Ref300N-23C biofilm cultures (2.9 × 10^5^ *nirK* cp/ng DNA) (Figs. 3A and 3B). All these results showed that strain GP59 was favored in the ASW medium with increasing concentrations of NaCl, which was the opposite for strain NL23 in these cultures.

### Cultivation of the original biofilm in the IO medium

The important decrease of strain NL23 and the enrichment of strain GP59 in the Ref300N-23C biofilm cultures suggest that the switch from the continuous-operating mode that prevailed in the Biodome reactor to the batch mode in our biofilm cultures could have generated these specific changes in the *H. nitrativorans* and *M. nitratireducenticrescens* populations. Another factor that could have influenced these changes is the ASW medium that we used instead of the Instant Ocean (IO) from Aquatic systems Inc. used by the Biodome (Table S1). The dispersed cells of the OB were therefore cultivated in the IO medium, under the same conditions than the Ref300N-23C biofilm cultures (performed in parallel). The NO_3_^−^ and NO_2_^−^ dynamics in the Ref300N-23C biofilm cultures were similar to those reported in Fig. 1A. The denitrification rates were calculated at 1.89 mM-NO_x_ h^−1^, and the specific denitrification rates corresponded to 0.0684 mM-NO_x_ h^−1^ mg-protein^−1^ (Table 3). In the IO biofilm cultures, the denitrification rates (0.76 mM-NO_x_ h^−1^) were 2.5 lower than in the Ref300N-23C biofilm cultures (Table 3). However, because less biomass developed in the IO biofilm cultures (Table 3), its specific denitrification rates (0.0661 mM-NO_x_ h^−1^ mg-protein^−1^) were the same as in the Ref300N-23C biofilm cultures.

As before, PCR-DGGE migration profiles (Fig. S5) and qPCR assays showed the decrease in strain NL23 concentrations during the five carrier transfers in the Ref300N-23C biofilm cultures. Surprisingly, the DGGE band corresponding to strain NL23 was still present in the IO biofilm cultures (Fig. S5). This result was confirmed by qPCR assays where the concentrations of strain NL23 in the IO biofilm cultures (Fig. 3A) were at similar levels (7.0 × 10^4^ *napA* cp/ng DNA) as in the OB (8.7 × 10^4^ *napA* copies/ng DNA). In the IO biofilm cultures, the concentrations of strain JAM1 and strain GP59 were both approximately one order of magnitude lower than those of strain NL23 (Fig. 3A).

### Cultivation of the original biofilm under oxic conditions and with NO${}_{2}^{-}$

The dispersed cells of the OB were cultivated under the same conditions of the Ref300N-23C biofilm cultures except that the cultures were incubated under oxic conditions. Growth was not impaired in these cultures, but the specific denitrification rates were 5 times lower than in the Ref300N-23C anoxic biofilm cultures (Table 3). Culturing the OB with a mix of 200 mg-NO_3_^−^-N/L and 200 mg-NO_2_^−^-N/L (here named 200-200N) generated biofilm cultures with denitrification rates 2.5 times lower than those in the Ref300N-23C biofilm cultures (Table 3). The proportions of *M. nitratireducenticrescens* strains JAM1/GP50 and strain NL23 in these two types of cultures were similar to the ones in the Ref300N-23C biofilm cultures (Figs. 3A and 3B).

## Discussion

The Montreal Biodome operated a continuous fluidized methanol-fed denitrification system between 1998 and 2006. After the termination of the system, our group took the opportunity to preserve carriers with the denitrifying biofilm in glycerol at −20 °C (Laurin et al., 2006). Our study showed that the bacterial community of the OB taken from the carriers could be revived by the colonization of new carriers, and could adapt to environment changes, which included the batch-operating mode, instead of the continuous mode, and the use of a homemade ASW medium, instead of the commercial IO medium used in the Biodome system. The resulting reference biofilm cultures could sustain denitrifying activities after a short exposure to various environmental changes such as a range of concentrations of NaCl (0% to 8%), NO_3_^−^ (6.4 to 214 mM), NO_2_^−^ (14.3 to 28.6 mM) and methanol (0% to 0.75%), and a range of pH (4 to 8) and temperature (4  °C to 36 °C). Denitrifying activities were inhibited when the biofilm cultures were exposed at pH 10 or with 1.5% methanol, and were strongly impacted by the presence of NO_2_^−^. Low pHs had a strong effect on the denitrification rates, with a 2.7-fold increase at pH 4.0 than at pH 8.0 in the original conditions. At the end of the assays (10–12 h), however, the pH in the medium increased to reach pH 8.0. Denitrification is an alkalization process that leads to the increase of pH in the medium (Lee & Rittmann, 2003). Phosphate used in ASW as buffering system explains the stabilization of the pH at around 8.0.

Cultivating the OB in ASW had a strong impact on the population of *H. nitrativorans* strain NL23. A decrease of three orders of magnitude was noticed in the levels of strain NL23 after 5 weeks in the Ref300N-23C biofilm cultures. However, this important decrease did not translate into impaired denitrification rates. On the opposite, the concentrations of *M. nitratireducenticrescens* in these biofilm cultures increased by two orders of magnitude during that time. We already reported in Geoffroy et al. (2018) that *H. nitrativorans* strain NL23 was displaced in ASW biofilm cultures by a new strain of *M. nitratireducenticrescens*, strain GP59, that can perform complete denitrification. The increase of concentrations of *M. nitratireducenticrescens* involved mainly strain GP59, which was at a very low level in the OB, but increased by three orders of magnitude in the Ref300N-23C biofilm cultures. Strain JAM1 stayed at the same levels in the OB and the Ref300N-23C biofilm cultures. The reasons why strain GP59 did not appear in first instance in the denitrification system of the Biodome are obscure.

In the second set of assays, a series of biofilm cultures were performed, in which the bacterial community of the OB was cultivated to different conditions for five weeks. The best denitrification performance occurred in the 900N-30C biofilm cultures (64.3 mM NO_3_^−^, 0.45% methanol, 30 °C), which allowed a 85% increase in the specific denitrification rates compared to the Ref300N-23C biofilm cultures. We did not try to cultivate the OB in low pHs because of the rapid fluctuations of the pH during the short exposure assays. The NaCl concentration was a critical factor for the persistence of *H. nitrativorans* strain NL23 in the biofilm cultures. In the ASW-modified media with low NaCl concentrations (0%, 0.5% and 1.0%), high levels of *H. nitrativorans* strain NL23 were found. Martineau, Mauffrey & Villemur (2015) showed that strain NL23 can sustain good growth and denitrifying activities up to 1% NaCl in planktonic pure cultures, but underwent substantial decrease in these features at 2% NaCl. In contrast, growth of *Methylophaga* spp. requires Na^+^ (Bowman, 2005). In the 0% NaCl biofilm cultures, low concentration of Na^+^ (0.06%; originating from NaNO_3_, Na_2_HPO_4_ and NaOH, Table S1) may have impaired the growth of *M. nitratireducenticrescens*. Consequently, strain *H. nitrativorans* NL23 may have better competed against the *Methylophaga* strains in these cultures. On the opposite, the rapid processing of NO_3_^−^ by *M. nitratireducenticrescens* strain JAM1 (Mauffrey, Martineau & Villemur, 2015) during the acclimation of the bacterial community to new culture conditions in the Ref300N-23C biofilm cultures (containing 2.75% NaCl) with transient accumulation of NO_2_^−^ may have impaired the growth of *H. nitrativorans* strain NL23. The adverse environment for strain NL23 would have favored the immergence of strain GP59 that took over strain NL23 in completing the full denitrification pathway.

An interesting result was to find the persistence of strain NL23 in the biofilm cultivated under the same conditions than the Ref300N-23C biofilm cultures but with the commercial IO medium. The concentrations of *M. nitratireducenticrescens* strains JAM1/GP59 and *H. nitrativorans* strain NL23 were similar in the IO biofilm cultures. Although the IO biofilm cultures generated less biomass than the Ref300N-23C biofilm cultures, the specific denitrification rates were the same between the two types of cultures, suggesting similar dynamics of NO_3_^−^ processing by both bacterial communities. The reason of this fundamental difference has to be found in the composition of the seawater medium. Based on limited information on the composition of the IO medium, a difference that might impair the growth of strain NL23 is the lack of bicarbonate and carbonate in the ASW medium. *Hyphomicrobium* spp. use the serine pathway for the C1 assimilation, which requires one molecule of CO_2_ and two molecules of formaldehyde in each cycle forming a three-carbon intermediate, while in *Methylophaga* spp. the ribulose monophosphate cycle requires three molecules of formaldehyde (Anthony, 1982).

The co-occurrence of *Methylophaga* spp. and *Hyphomicrobium* spp. has also been reported in two other denitrifications systems. Osaka et al. (2008) studied a laboratory-scale continuously stirred tank reactor with synthetic wastewater and sludge adapted to denitrification conditions. Among the tested assays, the reactor was fed with methanol and acclimated with increasing concentrations of NaCl. This reactor could perform high levels of NO_3_^−^ removal at up to 3% NaCl, but a drastic decrease in the removal efficiency occurred at 4% NaCl. Further increases in NaCl concentrations failed to get denitrifying activities in the reactor. Co-occurrence of *Methylophaga* spp. and *Hyphomicrobium* spp. was revealed in cloned 16S rRNA gene libraries derived from the 0% and 4% NaCl synthetic wastewater media, with respectively 35% and 20% of the 16S clones affiliated to *Hyphomicrobium* spp., and 8% and 11% affiliated to *Methylophaga* spp. The other denitrification system is a methanol-fed fluidized-bed continuous denitrification system treating a marine aquarium in Helsinki, Finland (Rissanen et al., 2016). In this system, the biofilm developed on oolitic sand. *Methylophaga* spp. and *Hyphomicrobium* spp. were found in high proportions although high variations occurred between samples taken at different years. All these studies including ours showed the importance of these two taxa in marine denitrification processes. Our study demonstrated how these two taxa co-evolved in biofilm cultures subjected to environmental changes.

## Conclusions

The denitrifying biofilm taken from the Biodome denitrification system, which was frozen for many years, can be revived and cultivated at laboratory scale without the loss of denitrifying activities. The resulting reference biofilm cultures can sustain for a short period of time (2-5 days) changes in NaCl, NO_3_^−^, NO_2_^−^ and methanol concentrations, and in pH and temperature. Inhibition of the denitrifying activities occurred at high pH (10) and high methanol concentrations (1.5%). The biofilm cultures that resulted in the highest specific denitrification rates were those cultivated for 5 weeks with 64.3 mM NO_3_^−^, 0.45% methanol at 30 °C. The OB can sustain growth and denitrifying activities in ASW with low concentrations of NaCl, but showed poor developmental growth in ASW with 5% and 8% NaCl. Although lower biomass developed in the biofilm cultures cultivated in the commercial IO medium, specific denitrification rates similar to the ones of the reference biofilm cultures (ASW medium) were observed. Important changes in the co-occurrence of the populations of *M. nitratireducenticrescens* and *H. nitrativorans* were observed in these biofilm cultures when compared to the OB. In the ASW biofilm cultures, substantial growth of a subpopulation of *M. nitratireducenticrescens* (strain GP59 as representative) with full denitrification capacity displaced *H. nitrativorans* strain NL23. Strain NL23 however persisted in biofilm cultures cultivated in ASW with low concentration of NaCl, but also in the ones cultivated in the IO medium. This study shows the plasticity of the denitrifying biofilm to adapt to different conditions. Knowing the dynamics of the principal actors in the denitrifying biofilm in response to environmental changes will allow to better predict the behavior of a denitrification system in response to changes in the operating mode. For instance, as the mature biofilm cultures were able to denitrify under low or high NaCl concentrations, this suggests that the Biodome denitrification system could have sustained high variations in salt concentrations.

##  Supplemental Information

10.7717/peerj.7497/supp-1 Supplemental Information 1 Raw data for Figs. 1 to 3 and Table 3 Figure 1. Nitrate, nitrite and NOx concentrations in the Ref300N-23C biofilm cultures. Concentrations in the original biofilm (OB) and in the Ref300N-23°C biofilm cultures of Methylophaga nitratireducenticrescens (strains JAM1/GP59) and Hyphomicrobium nitrativorans strain NL23 by qPCR.
Figure 2. Denitrification rates determined in the Ref 300N-23°C biofilm cultures exposed for short period to different conditions, and the calculation of the relative denitrification rates with statistical analysis.
Figure 3. Concentrations in the different biofilm cultures of Methylophaga nitratireducenticrescens (strains JAM1/GP59, strain JAM1, strain GP59) and Hyphomicrobium nitrativorans strain NL23 by qPCR with statistical analysis.
Table 3. Denitrification rates and the concentrations of protein measured in the different biofilm cultures, and the calculation of the relative specific denitrification rates with statistical analysis.

10.7717/peerj.7497/supp-2 Supplemental Information 2 Supplemental Figures S1 to S5 Figure S1. Development of the reference biofilm cultures (Ref300N-23C)
Figure S2. Schematic of the conditions used to test the denitrification capacity of the Ref300N-23C biofilm cultures
Figure S3. Schematic of the procedures used to derive the biofilm cultures cultivated under different environmental conditions
Figure S4. Bacterial community profiles derived by PCR-DGGE of the biofilm cultures cultivated in ASW with different NaCl concentrations
Figure S5. Bacterial community profiles of the Ref300N-23C biofilm cultures and the IO biofilm cultures by PCR-DGGE

10.7717/peerj.7497/supp-3 Supplemental Information 3 Composition of natural seawater, the INSTANT OCEAN brand salt and the artificial seaeater (ASW) used in this study

10.7717/peerj.7497/supp-4 Supplemental Information 4 Supplemental Tables S2 to S3 Table S2. Primers used in this study
****Table S3. Denitrifying activities of the Ref300N-23C biofilm cultures exposed for short period under specific conditions

## Additional Information and Declarations

### Data Availability

The following information was supplied regarding data availability: The raw data for Figs. 1 to 3 are available in File S1.
