# Supplementary material for: Dynamics of a methanol-fed marine denitrifying biofilm: 1-Impact of environmental changes on the denitrification and the co-occurrence of Methylophaga nitratireducenticrescens and Hyphomicrobium nitrativorans
Source: PeerJ. 2019 Aug 13;7:e7497. doi: 10.7717/peerj.7497 (PMC6697038; doi:10.7717/peerj.7497)
Supplement: Supplemental Information 2 — Figure S1. Development of the reference biofilm cultures (Ref300N-23C) Figure S2. Schematic of the conditions used to test the denitrification capacity of the Ref300N-23C biofilm cultures Figure S3. Schematic of the procedures used to derive the biofilm cultures cultivated under different environmental conditions Figure S4. Bacterial community profiles derived by PCR-DGGE of the biofilm cultures cultivated in ASW with different NaCl concentrations Figure S5. Bacterial community profiles of the Ref300N-23C biofilm cultures and the IO biofilm cultures by PCR-DGGE [file peerj-07-7497-s002.pdf]

Growth conditions: Ref300N-23C (ASW, 300 mg-NO_3^--N/L, 2.75% NaCl, 23°C)

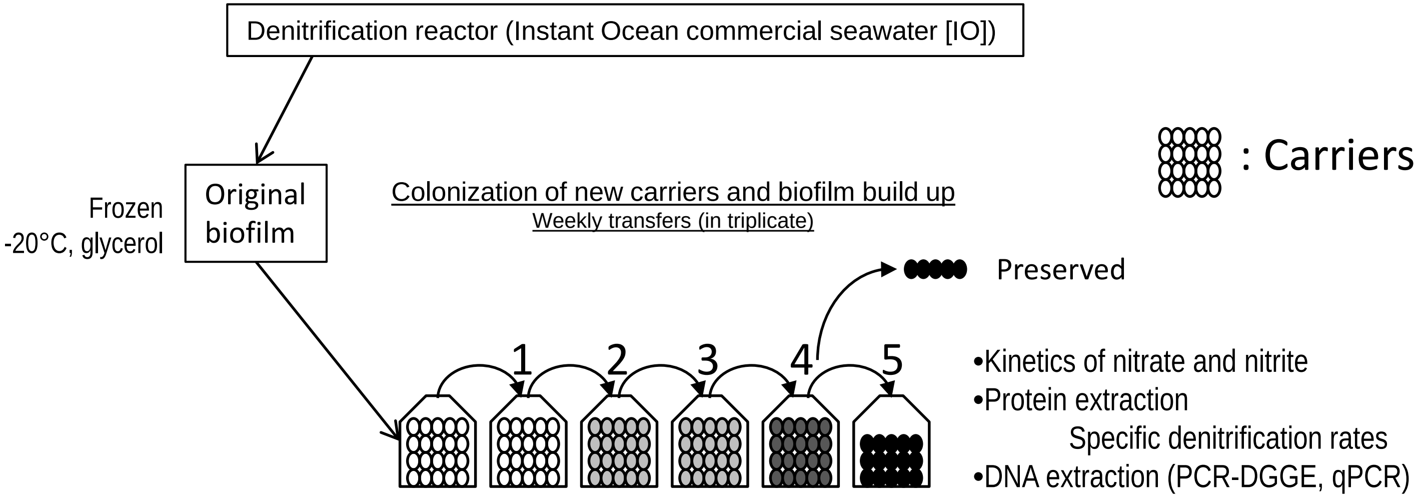

Figure S1. Development of the reference biofilm cultures (Ref300N-23C)

The original biofilm was thawed, scraped from the carriers and distributed to vials containing the ASW medium supplemented with 300 mg $NaNO_3$-N/L and 0.15% methanol and 20 free carriers. These carriers were transferred 5 times in fresh medium. Concentrations of NO_3^- and NO_2^- were measured at regular intervals. Methanol and $NaNO_3$ were added when needed if NO_3^- was completely depleted during the week.

Figure S2. Schematic of the conditions used to test the denitrification capacity of the Ref300N-23C biofilm cultures

-The original biofilm was thawed, scraped from the carriers and distributed to vials containing the ASW medium supplemented with 300 mg $NaNO_3$-N/L and 0.15% methanol and 20 free carriers. These carriers were transferred 5 times in fresh medium. At the 5th, 10th, 15th and 20th carrier-transfers, series of three colonized carriers were distributed into other vials containing fresh culture medium and 17 new carriers. The vials were cultivated and the carriers transferred as before.

-At the 10th, 12th, 15th and 20th carrier-transfers, series of 15 freshly colonized carriers were transferred into vials containing the prescribed medium to assess the impact in varying the NO_3^-, NO_2^- and methanol concentrations or the oxic conditions on the denitrifying activities.

-At the 25th transfer, series of 15 freshly colonized carriers were transferred into vials containing the prescribed medium to assess the impact in varying the pH, the temperature and the NaCl concentrations on the denitrifying activities. The biofilm was cultivated for two days in these prescribed conditions before the carriers to be transferred in fresh prescribed medium.

-Kinetics of NO_3^- and NO_2^- were monitored at regular intervals. Protein and DNA extractions were then performed from the biofilm and the suspended biomass.

Figure S2

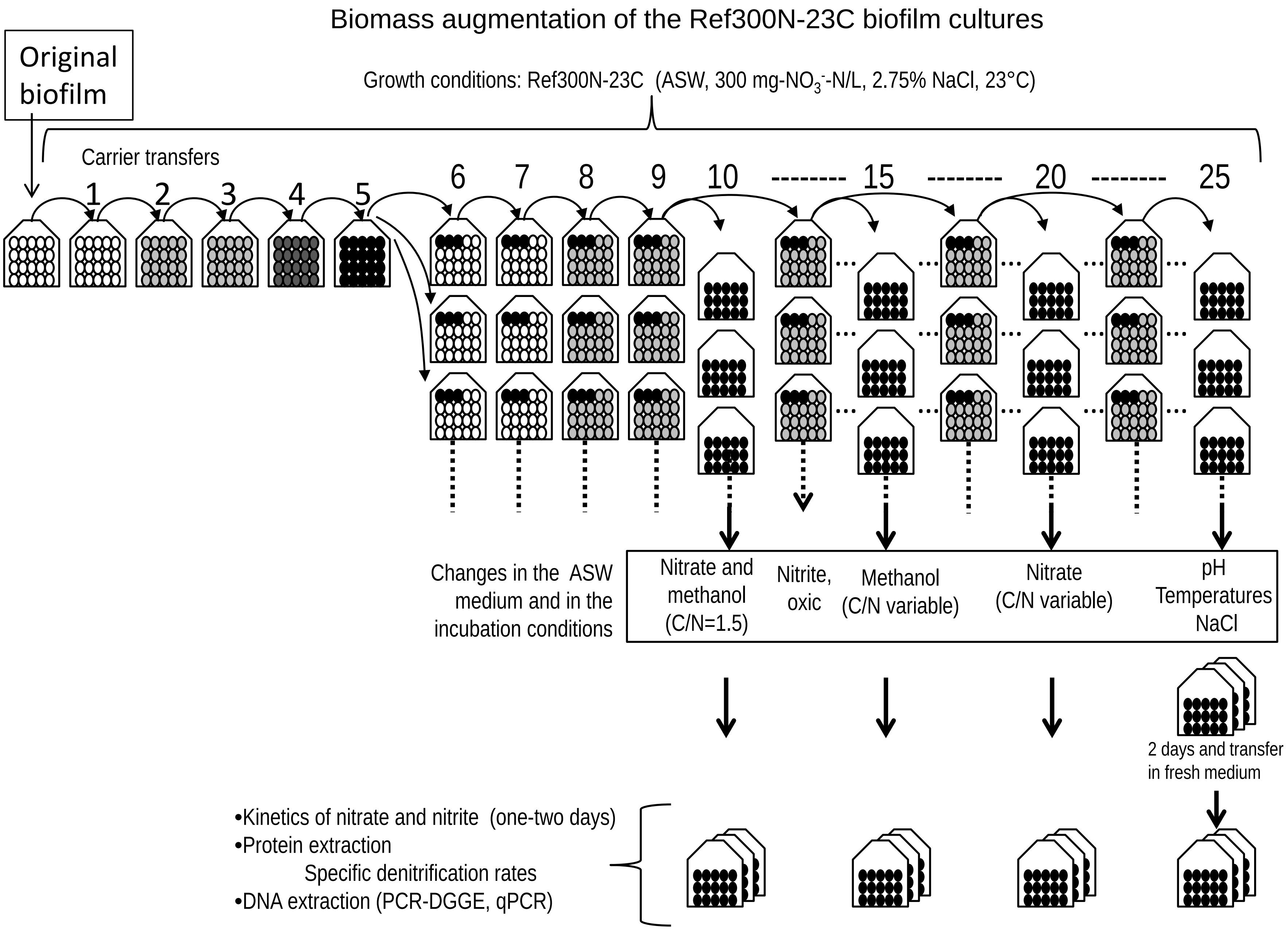
Biomass augmentation of the Ref300N-23C biofilm cultures
Original biofilm
Growth conditions: Ref300N-23C (ASW, 300 mg-NO_3^--N/L, 2.75% NaCl, 23°C)
Carrier transfers
1
2
3
4
5
6
7
8
9
10
-------- 15
-------- 20
-------- 25
Changes in the ASW medium and in the incubation conditions
Nitrate and methanol (C/N=1.5)
Nitrite, oxic
Methanol (C/N variable)
Nitrate (C/N variable)
pH
Temperatures
NaCl
2 days and transfer in fresh medium
•Kinetics of nitrate and nitrite (one-two days)
•Protein extraction
Specific denitrification rates
•DNA extraction (PCR-DGGE, qPCR)

Figure S3. Schematic of the procedures used to derive the biofilm cultures cultivated under different environmental conditions

The original biofilm was thawed, scraped from the carriers and distributed to vials containing the ASW medium supplemented with prescribed concentrations of NO_3^-, NO_2^-, methanol and NaCl, or containing the IO medium (Table 2), and 20 free carriers. The vials were incubated at the prescribed temperatures and at pH 8 under anoxic or oxic conditions (Table 2). In average once a week, the carriers were transferred five times into fresh prescribed medium and incubated in the same conditions. For the 2.75-5%NaCl assay, the first five transfers were incubated in the same conditions of Ref300N-23C cultures, then the three subsequent transfers were incubated in ASW medium with 5% NaCl. NO_3^- and NO_2^- concentrations were measured in each one or two days. Methanol and $NaNO_3$ were added when needed if NO_3^- was completely depleted during the week. DNA was extracted from the biomass for PCR-DGGE and qPCR assays. During the 5th carrier-transfer cultures (or the 8th for the 2.75-5%NaCl and the 200/200N biofilm cultures), 15 carriers from each vial were transferred into their respective fresh medium, and concentrations of NO_3^- and NO_2^- were measured at regular intervals. Protein content in the whole vials was then measured.

Figure S3

BIOFILM CULTURE CONDITIONS

5 transfers (all under anoxic conditions)

- Ref300N-23C (ASW, 300 mg-NO_3^--N/L, 2.75% NaCl, 23°C): Reference biofilm cultures
- 300N-30C (ASW, 300 mg-NO_3^--N/L, 2.75% NaCl, 30°C)
- 900N-23C (ASW, 900 mg-NO_3^--N/L, 2.75% NaCl, 23°C)
- 900N-30C (ASW, 900 mg-NO_3^--N/L, 2.75% NaCl, 30°C)
- 0%NaCl (ASW, 300 mg-NO_3^--N/L, 0% NaCl, 23°C)
- 0.5%NaCl (ASW, 300 mg-NO_3^--N/L, 0.5% NaCl, 23°C)
- 1%NaCl (ASW, 300 mg-NO_3^--N/L, 1.0% NaCl, 23°C)
- 5%NaCl (ASW, 300 mg-NO_3^--N/L, 5% NaCl, 23°C)
- 8%NaCl (ASW, 300 mg-NO_3^--N/L, 8% NaCl, 23°C)
- IO (Instant Ocean, 300 mg-NO_3^--N/L, 23°C)
- 200/200N (ASW, 200 mg-NO_3^--N/L, 200 mg-NO_2^--N/L, 2.75% NaCl, 23°C) (8 transfers)

5 transfers under oxic conditions (Erlenmeyer flasks)

- 300N-23C oxic (ASW, 300 mg-NO_3^--N/L, 2.75% NaCl, 23°C)

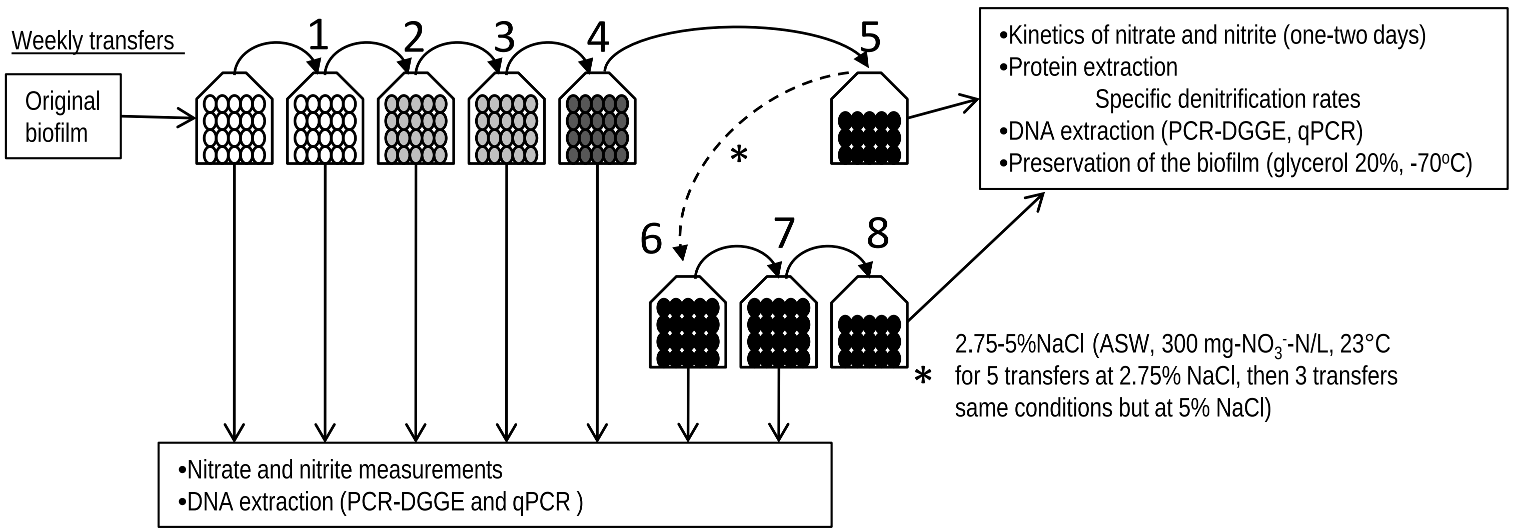

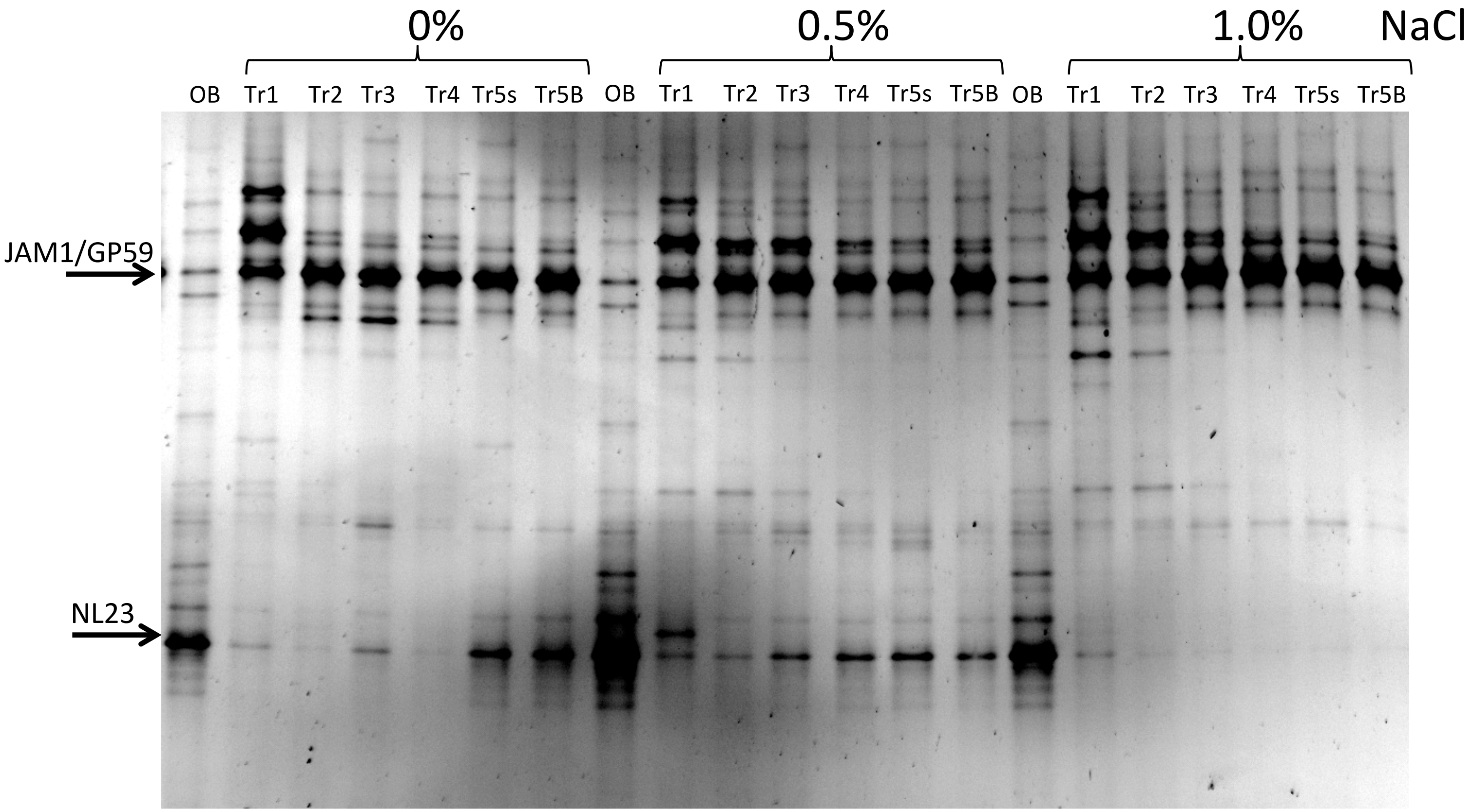

Figure S4. Bacterial community profiles derived by PCR-DGGE of the biofilm cultures cultivated in ASW with different NaCl concentrations

The original biofilm (OB) was cultivated in the ASW medium composed of 0%, 0.5% and 1.0% NaCl. Carriers were transferred 5 times. DNA extraction was performed at the end of the carrier-transfer cultures. PCR–DGGE migration profiles (8% polyacrylamide DGGE with a 30% to 70% denaturant gradient) of 16S rRNA gene sequences were derived from the OB, the suspended biomass of the 1st to the 5th carrier-transfers (Tr1 to Tr5S), and the carrier biofilm after the 5th carrier-transfer (Tr5B). Triplicates produced the same profiles.

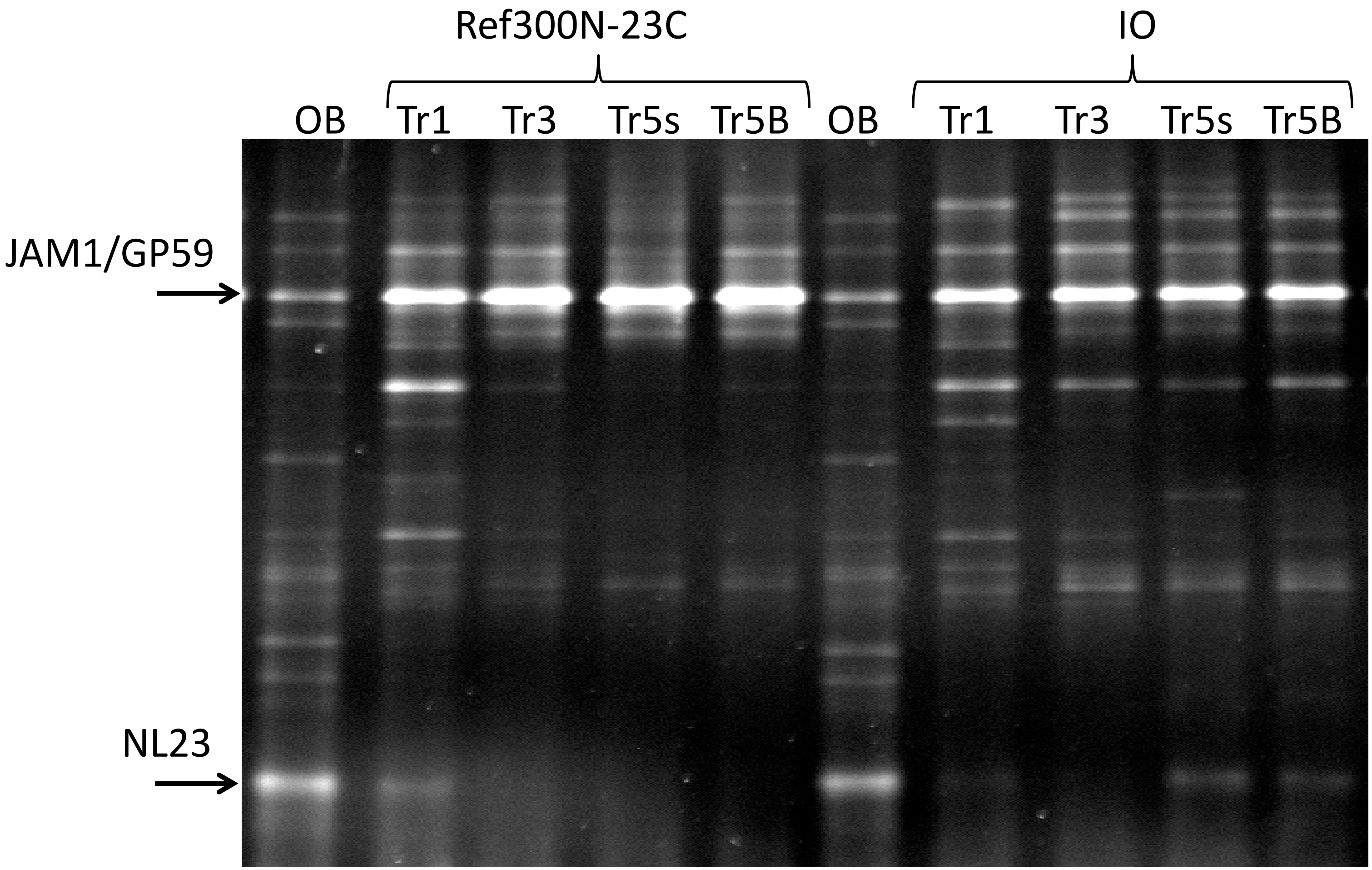

Figure S5. Bacterial community profiles of the Ref300N-23C biofilm cultures and the IO biofilm cultures by PCR-DGGE

The original biofilm (OB) was cultivated in the IO medium under the same conditions of the Ref300N-23C biofilm cultures (ASW). DNA was extracted from the OB, from the suspended biomass after the 1st, 3rd and 5th carrier-transfers, and from the carrier biofilm after the 5th carrier-transfers (Tr5B). 16S rRNA gene sequences were PCR amplified and segregated on PCR–DGGE (8% polyacrylamide DGGE with a 30% to 70% denaturant gradient). Triplicates produced the same profiles.
