# Supplementary material for: Dynamics of a methanol-fed marine denitrifying biofilm: 1-Impact of environmental changes on the denitrification and the co-occurrence of Methylophaga nitratireducenticrescens and Hyphomicrobium nitrativorans
Source: PeerJ. 2019 Aug 13;7:e7497. doi: 10.7717/peerj.7497 (PMC6697038; doi:10.7717/peerj.7497)
Supplement: Supplemental Information 4 — Table S2. Primers used in this study Table S3. Denitrifying activities of the Ref300N-23C biofilm cultures exposed for short period under specific conditions [file peerj-07-7497-s004.docx]

**Table S2. Primers used in this study**

Sequence (5' - 3') Th length Comments

°C bp

16S ribosomal RNA genes

341f * CCTACGGGAGGCAGCAG 55 ~250 DGGE

534r ATTACCGCGGCTGCTGG

*narG1* (strain JAM1/GP59; locus Q7A_446). Nitrate reductase subunit alpha (Nar1 system)

narG-JAM1_G ATGACAAGATCGTGCGTTCT 57 664 Standard

narG-JAM1_D GGTGTACGGGTCATTGGTAAG

narG1313f-JAM1 AGCCCACATCGTATCAAGCA 61 149 qPCR

narG1461r-JAM1 CCACGCACCGCAGTATATTG

*napA* (strain NL23, locus tag W911_13875) periplasmic nitrate reductase

napA1171f-NL23 TACAACGTCCACCTGCTGAC 61 375 Standard

napA1846r-NL23 TCCGCTTCGTGGTTTTCGTA

napA1415f-NL23 AGGACGGGCGGATCAATTTT 61 131 qPCR

napA1526r-NL23 CGGATATGCATCGGACACGA

*tagH* (strain JAM1, locus tag: Q7A_1110). Teichoic acid export ATP-binding protein

tagH Forward ccgtcatttcgcttcaagat 55 711 Standard

tagH Reverse tcatggctttttcagccttt

qtagH Forward gttgcaaggctatagtcggagt 55 119 qPCR

qtagH Reverse tggtacgcattccagatgaata

*nirK* (strain GP59, locus tag: CDW43_15165). Copper containing nitrite reductase

nirK Forward cgttcaatacatggggtaaagg 55 1134 Standard

nirK Reverse tggggcacagtgataaacaa

qnirK Forward aagtcggtaaagtagccgttga 55 138 qPCR

qnirK Reverse tctccatcgtcatttgaacaac

* GC tail added at the 5' end CGCCCGCCGCGCGCGGCGGGCGGGGCGGGGGCACGGGGGG (Muyzer *et al*., 1993).

Locus tags are from the genomes of *M. nitratireducentricrescens* JAM1 (GenBank accession number CP003390.3) and *H. nitrativorans* NL23 (CP006912.1).

Muyzer G, de Waal EC, Uitterlinden AG. Profiling of complex microbial populations by denaturing gradient gel electrophoresis analysis of polymerase chain reaction-amplified genes coding for 16S rRNA. Appl Environ Microbiol. 1993, 59:695-700.

**Table S3. Denitrifying activities of the Ref300N-23C biofilm cultures exposed for short period under specific conditions**

Name Denitrification Relative Protein Specific

rates denitrification concentration denitrification

rates ¶ rates

mM-NO_x_ mg vial^-1^ mM-NO_x_ h^-1^

Tested conditions h^-1^ vial^-1^ mg-protein^-1^

NO_3_^-^ and methanol (C/N=1.5)

*300_N-NO3_-0.15%_MeOH_**  1.84 (0.01) 1.00 (0.00) 27.3 (2.1) 0.0678 (0.0050)

600_N-NO3_-0.3%_MeOH_ 2.91 (0.05) 1.58 (0.03) 28.3 (4.5) 0.1042 (0.0171)

900_N-NO3_-0.45%_MeOH_ 2.88 (0.11) 1.56 (0.06) 24.7 (2.2) 0.1171 (0.0010)

1500_N-NO3_-0.75%_MeOH_ 2.51 (0.19) 1.36 (0.10) 26.9 (6.8) 0.0971 (0.0207)

3000_N-NO3_-1.5%_M eOH_ negligible na nd na

Methanol (C/N variable)

0%_MeOH_ 0.22 (0.02) 0.15 (0.01) 30.1 (5.2) 0.0071 (0.0007)

0.05%_MeOH_ 1.10 (0.10) 0.72 (0.07) 31.0 (6.8) 0.0364 (0.0057)

*0.15%_MeOH_**  1.52 (0.02) 1.00 (0.01) 27.7 (2.8) 0.0550 (0.0057)

0.5%_MeOH_ 1.61 (0.07) 1.06 (0.05) 28.9 (1.5) 0.0557 (0.0050)

NO_3_^-^ (C/N variable)

90_N-NO3_ 0.81 (0.02) 0.66 (0.02) 32.1 (0.6) 0.0257 (0.0007)

*300_N-NO3_**  1.24 (0.01) 1.00 (0.01) 31.3 (1.0) 0.0400 (0.0014)

900_N-NO3_ 2.15 (0.04) 1.74 (0.03) 32.2 (1.2) 0.0671 (0.0014)

3000_N-NO3_ 0.57 (0.01) 0.46 (0.01) 28.5 (1.4) 0.0200 (0.0014)

pH

pH4 2.23 (0.13) 2.75 (0.15) 30.4 (1.5) 0.0736 (0.0071)

pH6 2.09 (0.23) 2.59 (0.28) 35.1 (3.2) 0.0600 (0.0071)

pH8* 0.81 (0.04) 1.00 (0.05) 32.1 (1.7) 0.0250 (0.0029)

pH10 negligible na nd na

Temperature

5°C 0.22 (0.00) 0.27 (0.00) 38.2 (1.1) 0.0057 (0)

15°C 0.65 (0.01) 0.80 (0.01) 33.6 (1.0) 0.0193 (0.0007)

23°C* 0.81 (0.04) 1.00 (0.05) 32.1 (1.7) 0.0250(0.0029)

30°C 1.24 (0.13) 1.52 (0.16) 33.6 (2.2) 0.0371 (0.0043)

36°C 1.31 (0.01) 1.62 (0.01) 31.5 (0.5) 0.0414 (0.0007)

NaCl

0%_NaCl_ 0.85 (0.01) 1.05 (0.01) 30.2 (0.9) 0.0286 (0.0007)

1%_NaCl_ 0.92 (0.04) 1.13 (0.05) 36.5 (0.7) 0.0250 (0.0007)

*2.75%_NaCl_** 0.81 (0.04) 1.00 (0.05) 32.1 (1.7) 0.0250 (0.0029)

5%_NaCl_ 0.89 (0.04) 1.10 (0.05) 35.0 (1.3) 0.0257 (0.0021)

8%_NaCl_ 1.01 (0.01) 1.24 (0.02) 37.8 (2.7) 0.0264 (0.0021)

NO_2_^-^

400_N-NO2_ 0.15 0.09 nd

200_N-NO2_ 0.26 0.15 nd

200_N_-_NO3_/200_N-NO2_ 0.51 0.31 nd

See Table 1 and Material & Methods for nomenclature and culture conditions. Average of triplicates cultures except for the nitrite assays (single culture). Values between parentheses: standard deviation.

*: Ref300N-23C biofilm cultures. nd: not detected. na: not applicable.

¶Relative denitrification rates were normalized by the average denitrification rates of the Ref300N-23C biofilm cultures.
